# Lymph node ratio prognosticates overall survival in patients with stage IV colorectal cancer

**DOI:** 10.1007/s10151-024-02984-6

**Published:** 2024-08-23

**Authors:** K. Naidu, P. H. Chapuis, L. Connell, C. Chan, M. J. F. X. Rickard, K-S. Ng

**Affiliations:** 1 Colorectal Surgery Unit, Concord Hospital, Concord, NSW 2139 Australia; 2 Concord Institute of Academic Surgery, Concord Repatriation General Hospital, Building 20, Level 1, Hospital Road, Concord, NSW 2139 Australia; 3 Concord Clinical School, Clinical Sciences Building, University of Sydney, Concord Repatriation General Hospital, Concord, NSW 2139 Australia; 4 Department of Anatomical Pathology, Concord Repatriation General Hospital, Concord, NSW 2139 Australia

**Keywords:** Lymph node ratio, LNR, Lymphadenectomy and metastatic colorectal cancer

## Abstract

**Background:**

Lymph node ratio (LNR) is suggested to address the shortcomings of using only lymph node yield (LNY) or status in colorectal cancer (CRC) prognosis. This study explores how LNR affects survival in patients with metastatic colorectal cancer (mCRC), seeking to provide clearer insights into its application.

**Methods:**

This observational cohort study investigated stage IV patients with CRC (1995–2021) who underwent an upfront resection of their primary tumour at Concord Hospital, Sydney. Clinicopathological data were extracted from a prospective database, and LNR was calculated both continuously and dichotomously (LNR of 0 and LNR > 0). The primary endpoint was overall survival (OS). The associations between LNR and various clinicopathological variables were tested using regression analyses. Kaplan–Meier and Cox regression analyses estimated OS in univariate and multivariate survival models.

**Results:**

A total of 464 patients who underwent a primary CRC resection with clear margins (mean age 68.1 years [SD 13.4]; 58.0% M; colon cancer [*n* = 339,73.1%]) had AJCC stage IV disease. The median LNR was 0.18 (IQR 0.05–0.42) for colon cancer (CC) resections and 0.21 (IQR 0.09–0.47) for rectal cancer (RC) resections. A total of 84 patients had an LNR = 0 (CC = 66 patients; RC = 18 patients). The 5-year OS for the CC cohort was 10.5% (95% CI 8.7–12.3) and 11.5% (95% CI 8.4–14.6) for RC. Increasing LNR demonstrated a decline in OS in both CC (*P* < 0.001) and RC (*P* < 0.001). In patients with non-lymphatic dissemination only (LNR = 0 or N0 status), there was better survival compared with those with lymphatic spread (CC aHR1.50 [1.08–2.07;*P* = 0.02], RC aHR 2.21 [1.16–4.24;*P* = 0.02]).

**Conclusions:**

LNR is worthy of consideration in patients with mCRC. An LNR of 0 indicates patients have a better prognosis, underscoring the need for adequate lymphadenectomy to facilitate precise mCRC staging.

## Introduction

In patients with metastatic colorectal cancer (mCRC) who undergo a primary tumour resection with clear margins, a distinct observation is noted in that the lymph node yield (LNY) from resected specimens is frequently substandard (i.e. fewer than 12 nodes are retrieved), when compared with their non-metastatic counterparts [1–4]. This observation may be embedded in the perception that once distant metastatic disease has occurred, addressing LN evaluation is futile [5].

While it is true that the presence of distant metastases represents the most advanced stage of the disease, it may not negate the importance of LN evaluation in mCRC management. Understanding the extent of regional LN involvement may still be relevant, as LN status (even in the presence of distant metastases) may potentially reflect differences in disease behaviour, tumour aggressiveness and overall survival. Similarly, precise LN evaluation might help inform decisions on the use of post-operative therapy, targeted treatments and the frequency of follow-up care [3].

Lymph node ratio (LNR), calculated as the number of metastatic LNs divided by the total number of examined nodes [6], may be a useful parameter that incorporates information relating to both LN status and LNY. The prognostic significance of LNR in stage III CRC has been well documented, with previous studies demonstrating that those with higher LNR values tend to have poorer survival [6–8]. Notably, LNR offers a more distinct prognostic differentiation when compared with the sole reliance on a simple count of positive nodes [7, 8]. However, the impact of LNR on mCRC patients’ survival remains unclear. In particular, patients’ survival with an LNR of 0 (i.e. node negative, or N0, metastatic disease) have not been sufficiently explored in literature.

In mCRC patients with an LNR of 0 (i.e. N0 status), the absence of metastatic LNs coexists with metastatic spread of disease through other routes such as haematogenous or transcoelomic spread. Intuitively, these patients are presumed to have a better prognosis due to a potentially decreased tumour burden. Investigating the survival implications of dichotomizing LNR (LNR of 0 and LNR > 0) may refine our understanding of patients with an LNR of 0. Additionally, such a dichotomization may provide insight into the need to perform an adequate lymphadenectomy in patients with mCRC undergoing primary tumour resection (i.e. upfront surgery).

This study aims to examine if LNR is an independent predictor of overall survival (OS) in patients with mCRC following resection of the primary tumour. We analysed LNR both as a continuous variable and dichotomously (LNR of 0, LNR > 0), hypothesising that the rising LNR correlates with poorer survival, while LNR = 0 would be associated with better outcome.

## Materials and methods

**Fig. 1:**
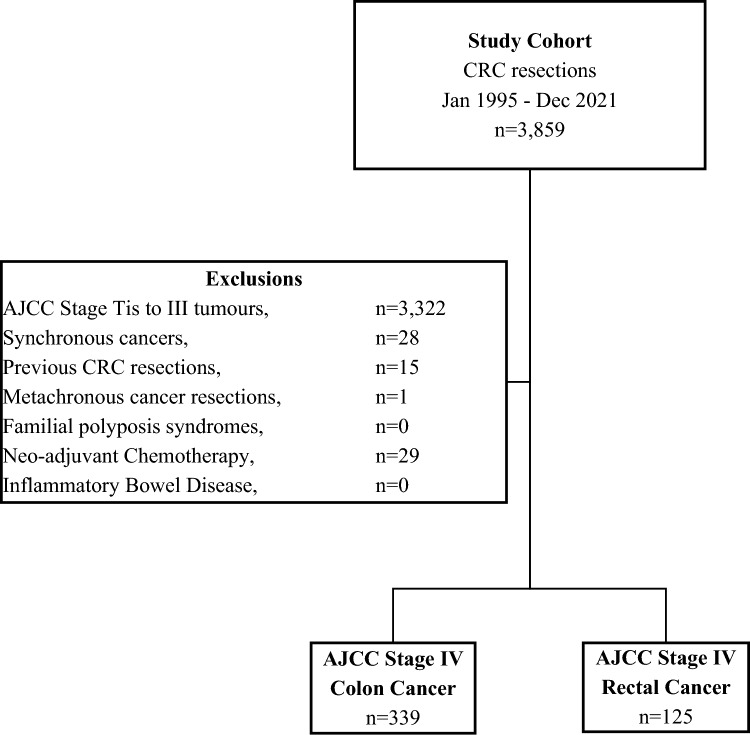
Flow diagram of cohort definition

A cohort study of consecutive patients who had an upfront resection for a newly diagnosed AJCC stage IV colorectal adenocarcinoma at Concord Hospital, Sydney, between January 1995 and December 2021, was performed. The study population was drawn from a prospectively maintained institutional database, which has been in continual existence since January 1971. Patients with in situ neoplasia, AJCC stage I to III CRC, polyposis coli, inflammatory bowel disease and/or synchronous or metachronous CRC were excluded (Fig. 1). The study received ethical approval from the Sydney Local Health District Ethics Committee (2019/ETH07841).

### Pre-operative assessment

For patients planned for an elective operation, pre-operative tumour status was assessed in a multidisciplinary setting based on clinical examination, colonoscopy, computed tomography, ultrasonography, MRI and, more recently, selective positron emission tomography.

### Neo-adjuvant therapy

In more recent study periods, systemic neo-adjuvant chemotherapy (nCT) was introduced for patients with mCRCs. These patients were excluded from this study for several reasons. First, nCT has been shown to artefactually influence LNY [9], potentially confounding LNR’s impact on outcomes. Moreover, an upfront resection followed by post-operative treatment of synchronous metastases formed the mainstay of management during much of the study period and today remains a recognised strategy in the treatment of mCRC [2, 4, 10–13].

### Neo-adjuvant therapy in rectal cancer

For non-urgent rectal cancers located in the lower two-thirds of the rectum, a patient’s suitability for neo-adjuvant radiotherapy (short versus long-course with or without chemotherapy) was decided by multidisciplinary consensus based on: the patient’s individual needs, tumour location, clinical and radiological features, biopsy findings, metastasis extent, history of previous pelvic irradiation and the patient's fitness for operation.

## Surgical management

### Primary CRC

During the majority of the study period, the predominant approach to managing mCRC entailed upfront surgical resection of the primary CRC followed by post-operative chemotherapy. This strategy continues to be a recognized method in mCRC treatment [2, 4, 10–15]. For patients who underwent emergency primary CRC resection, the surgical indications were typically large bowel obstruction, perforation or bleeding.

A resection for a primary CRC was classified into three categories: standard, extended or a segmental operation. This classification was based on the extent of the vascular ligation of the tumour which consequently determined the extent of lymphadenectomy performed [16]. Standard resections included right hemicolectomy, left hemicolectomy, transverse colectomy, sigmoid colectomy, a Hartmann’s procedure, anterior resection and abdominoperineal excision (APER), wherein the operation involved the ligation of strategic vessels, such as the ileocolic artery, right colic artery (if present), middle colic vessels, ascending left colic artery and inferior mesenteric artery. An extended resection comprised either an extended right hemicolectomy or a subtotal colectomy, involving the ligation of a combination of the aforementioned vessels. For instance, in the case of an extended right hemicolectomy, the ligation includes the ileocolic artery, right colic artery (if present) and middle colic vessels. Lastly, segmental resections entailed the removal of the primary tumour without a dedicated vascular pedicle ligation, resulting in varying LNY [16]. Tumours between (and including) the caecum and rectosigmoid junction were defined as colonic. Rectal cancers were defined as those whose inferior edge was within 15 cm from the anal verge, measured by rigid sigmoidoscopy [17].

### Metastasectomy

For patients who underwent a metastasectomy, this was performed during the index operation or as a separate staged operation. The decision and timing hinged on factors including the clinical presentation of the primary cancer, the extent of metastatic spread, the patient’s fitness for surgery and response to post-operative chemotherapy.

In selected cases where metastasectomy was deemed unsuitable but the patient was symptomatic and had a good performance status, a palliative resection of the primary tumour and regional lymphadenectomy was performed followed by palliative chemotherapy, if appropriate.

### Pathology reporting and staging

Specialist pathologists examined resected specimens using a standard synoptic protocol, with pathology data coded by CC [16]. Adenocarcinomas including mucinous and signet ring types were analysed. Tumour staging followed the AJCC pTNM system [18].

### Standard clinicopathological variables

Clinical information, operative details, tumour pathology, treatment information and follow-up data were extracted from the database for analysis, as previously described [16]. Groupings for year of resection was also performed to acknowledge the potential changes in treatments approaches over time.

### Clinicopathological variable of interest—LNR

The LNR, which represents the ratio of involved LNs to the total number of LNs harvested, calculated, and documented as an additional variable. It was also dichotomised as LNR of 0 and LNR > 0. This dichotomisation aimed to identify patients with mCRC with metastatic spread exclusively through non-lymphatic pathways (i.e. LNR of 0).

### Surveillance

In patients with mCRC who underwent a synchronous metastasectomy and primary tumour resection, their follow-up mostly resembled that of individuals with non-metastatic disease [16, 19]. Generally, this involved clinical examinations and liver function tests every 3 months, along with serial carcinoembryonic antigen measurements for the first 2 years post-operation, followed by these evaluations every 6 months for an additional three years, if clinically appropriate. Imaging typically included annual computed tomography scans for the first 2 years, with subsequent imaging guided by clinical and biochemical findings. More recently, selective positron emission tomography imaging has also been utilized. Patients were monitored annually until death or December 2021, with less than 5% of patients being lost to follow-up. Colonoscopy was generally conducted at 1-year post-resection.

Patients with unresectable metastatic disease following the resection of the primary tumour were referred to medical oncology and palliative care services for consideration of palliative chemotherapy. In these patients, a personalised and less demanding follow-up protocol was often employed.

### Post-operative chemotherapy

A multidisciplinary team routinely considered post-operative chemotherapy for all patients with mCRC who underwent primary tumour resection. Factors such as age, patient preferences, comorbidities, adverse pathological features, social circumstances, and best practice guidelines were considered.

The chemotherapy regimens utilised varied but were in accordance with best practice at the time and for the most part were: bolus injections of 5-fluorouracil (5-FU) and leucovorin administered daily in 5-day blocks and repeated every month for 6 months, as per the Mayo Clinic regimen [20]; 5-FU and leucovorin were repeated weekly for six doses with a 2-week rest between, as per the Roswell Park regimen [21]. An alternate option includes semi-monthly  22 h 5-FU infusion with leucovorin [22] or modified oxaliplatin, leucovorin and 5-FU (FOLFOX) was administered every 2 weeks [23]. In some, oral capecitabine and intravenous oxaliplatin (CAPOX or XELOX) was considered for six cycles. In some palliative settings, duplet (i.e. leucovorin, 5-FU with either oxaliplatin [FOLFOX] or irinotecan [FOLFIRI]) or triplet (i.e. leucovorin, 5-FU, oxaliplatin and irinotecan [FOLFOXIRI]) chemotherapy was considered.

### Outcome measures

The primary outcome measure was OS. This was considered the most pragmatic measure of survival in this patient cohort.

Patient follow-up commenced from the date of resection. Follow-up times were censored at the last contact for patients who did not experience a terminal event up to December 2021 or were lost to follow-up or remained alive. The date of death was primarily obtained from the patient’s surgeon, family physician or hospital records, with occasional use of the national death registration system [16]. The cause of death was coded by PC based on the International Classification of Diseases-10.

## Statistical analysis

Continuous variables were reported as mean (standard deviation [SD]) for normally distributed variables and as median (interquartile range [IQR] or range) for non-normal distributions. Categorical variables were reported as frequencies and percentages. Linear regression tested association between LNR (as a continuous variable) and other clinico-pathological variables. Logistic regression tested association between dichotomised LNRs (LNR of 0 or LNR > 0) and other clinico-pathological variables. These regression models were performed for colon and rectal cancer separately, due to perceived differences in their outcomes, biological behaviour and overall surgical treatment approach. Survival estimates were modelled using the Kaplan–Meier function with log-rank test performed to determine difference in survival distributions.

The association between LNR and OS was assessed by Cox regression, performed for colon and rectal cancers separately. To adjust for confounding, all clinico-pathological variables that showed associations (*P* < 0.05) with LNR on their respective linear (for continuous LNR) and logistic (for dichotomised LNR) regression analyses were also included in multivariable models. To avoid collinearity, LNR considered as continuous and dichotomised variables, were entered into two separate models. *P* < 0.05 was considered significant. All analyses were performed using SPSS^®^ v.29 (IBM, New York, USA).

## Results

### Study population

A total of 3859 patients underwent primary CRC resection, including 464 with AJCC stage IV disease. Among these, 269 (58.0%) were male and the mean age was 68.1 years (SD 13.4). Of the total, 339 patients (73.1%) had colon cancer. A standard operation was performed in 306 patients with colon cancer and 125 patients with rectal cancer. Specifically, among the patients with colon cancer, there were 122 (39.9%) cases of right hemicolectomy, 4 (1.3%) cases of transverse colectomy, 13 (4.2%) cases of left hemicolectomy, 11 (3.6%) cases of sigmoid colectomy, 50 (16.3%) cases of Hartmann’s procedure, and 106 (34.6%) cases of anterior resection. In the rectal cancer group, there were 28 (22.4%) cases of Hartmann’s procedure, 70 (56.0%) cases of anterior resection and 27 (21.6%) cases of APER. The study population’s characteristics are summarized in Table 1.

**Table 1:** Comparison of clinicopathological factors between patients with colon and rectal cancer (*n* = 464)

Variables	*N* (%) or mean (SD) or median (range/ IQR)	Colon cancer (*n* [%], total = 339)	Rectal cancer (*n* [%], total = 125)
Gender			
Male	269(58.0)	191(56.3)	78(62.4)
Female	195(42.0)	148(43.7)	47(37.6)
Age (mean [SD]), years	68.1(13.4)	69.1(13.6)	65.4(12.6)
BMI (mean [SD]), kg/m^2^	27.1(5.5)	27.1(5.7)	27.0(5.0)
ASA grade			
I	68(14.7)	40(11.8)	28(22.4)
II	232(50.0)	164(48.4)	68(54.4)
III/IV	164(35.3)	135(39.8)	29(23.2)
Year of resection			
1995–1999	95(20.5)	61(18.0)	34(27.2)
2000–2004	95(20.5)	59(17.4)	36(28.8)
2005–2009	94(20.3)	68(20.1)	26(20.8)
2010–2014	82(17.7)	71(20.9)	11(8.8)
2015–2019	74(15.9)	64(18.9)	10(8.0)
2020 onwards	24(5.2)	16(4.7)	8(6.4)
Emergency operation			
No	387(83.4)	265(78.2)	122(97.6)
Yes	77(16.6)	74(21.8)	3(2.4)
Type of operation			
Segmental resection	3(0.6)	3(0.9)	–
Standard resection	431(92.9)	306(90.3)	125(100.0)
Extended resection	30(6.5)	30(8.8)	–
Operation modality			
Open	321(69.2)	229(67.6)	92(73.6)
Laparoscopy	143(30.8)	110(32.4)	33(26.4)
Blood loss (millilitres)			
≤ 500	428(92.2)	317(93.5)	111(88.8)
> 500	36(7.8)	22(6.5)	14(11.2)
Tumour location (colon)			
Right	145(42.8)	145(42.8)	
Left	194(57.2)	194(57.2)	
Tumour location (rectum)			
Upper	47(37.6)		47(37.6)
Mid	43(34.4)		43(34.4)
Lower	35(28.0)		35(28.0)
Neoadjuvant RT (rectal)			
No	108(86.4)	NA	108(86.4)
Yes	17(13.6)	NA	17(13.6)
Distant metastatic sites			
Liver or other(s)	352(82.3)	279(82.3)	103(82.4)
Liver and other(s)	75(16.3)	54(15.9)	21(16.8)
Distant metastasectomy			
No	374(80.6)	275(81.1)	99(79.2)
Yes	83(17.9)	58(17.1)	25(20.0)
Tumour size (median [range]), cm	5.0(0.6–15.0)	5.0(0.6–15.0)	5.0(0.6–10.0)
Tumour perforation			
No	413(89.0)	307(90.6)	106(84.8)
Yes	51(11.0)	32(9.4)	19(15.2)
Obstructing tumour			
No	402(86.6)	280(82.6)	122(97.6)
Yes	62(13.4)	59(17.4)	3(2.4)
T stage			
T1 and T2	13(2.8)	6(1.8)	7(5.6)
T3	262(56.5)	173(51.0)	89(71.2)
T4	189(40.7)	160(47.2)	29(23.2)
Histological type			
Mucinous/signet ring	47(10.1)	39(11.5)	8(6.4)
Non-mucinous/signet ring	417(89.9)	300(88.5)	117(93.6)
Histological differentiation			
Well or moderate	297(64.0)	212(62.5)	85(68.0)
Poor	167(36.0)	127(37.5)	40(32.0)
Histological grade			
Low or average	251(54.1)	184(54.3)	67(53.6)
High	213(45.9)	155(45.7)	58(46.4)
Lympho-vascular invasion			
No	160(34.5)	120(35.4)	40(32.0)
Yes	304(65.5)	219(64.6)	85(68.0)
Peri-neural invasion			
No	254(54.7)	188(55.5)	66(52.8)
Yes	210(45.3)	151(44.5)	59(47.2)
Lymph nodes – examined (median [range])	17(2–87)	17(2–87)	17(3–49)
Lymph node yield			
< 12	103(22.2)	72(21.2)	31(24.8)
≥ 12	361(77.8)	267(78.8)	94(75.2)
Lymph node ratio—positive nodes/ total nodes examined (median [IQR])	0.20(0.06–0.43)	0.18(0.05–0.42)	0.21(0.09–0.47)
Lymph node ratio (LNR [dichotomised])			
LNR of 0	84(18.1)	66(19.5)	18(14.4)
LNR > 0	380(81.9)	273(80.5)	107(85.6)

*BMI* body mass index, *ASA* American Society of Anesthesiology, *RT* radiotherapy, *NA* not applicable, *T stage*, tumour stage (part of AJCC TNM system)

Of the 464 stage IV patients, 286 (61.6%) had isolated liver metastases, while 96 (20.7%) had metastases to other organs (lung and/or brain) exclusively. In 75 patients (16.3%), metastatic disease affected both the liver and other organs. A metastasectomy was performed in 83 patients (17.9%). A total of 65 patients (78.3%) had a staged metastasectomy; of these, 50 (76.9%) were performed for synchronous metastatic disease and 15 (23.1%) for metachronous disease.

## LNR

Both colon and rectal cancer resection specimens showed a median LN count of 17 nodes (colon range: 2–87, rectal range: 3–49). Most resections yielded 12 or more nodes (Table 2). In colon cancer resections, a median of three positive nodes (range: 0–28) were found, while rectal cancer resections had a median of four involved nodes (range 0–44). This resulted in a median LNR of 0.18 (IQR 0.05–0.42) for colon cancer resections and 0.21 (IQR 0.09–0.47) for rectal cancer resections. Notably, 66 (19.5%) patients with colon cancer and 18 (14.4%) rectal cancer patients had an LNR of 0.

**Table 2:** Comparison of LNY between patients with colon and rectal cancer with LNR of 0 and LNR > 0 (*n* = 464)

Variables	*N* (%) or mean (SD) or median (range/IQR)	Colon cancer (total, 339)		Rectal cancer (total, 125)	
		LNR of 0 (*n* [%], total, 66)	LNR > 0 (*n* [%], total, 273)	LNR of 0 (*n* [%], total, 18)	LNR > 0 (*n* [%], total, 107)
Lymph node yield					
< 12	103(22.2)	16(24.2)	56(20.5)	9(50.0)	22(20.6)
≥ 12	361(77.8)	50(75.8)	217(79.5)	9(50.0)	85(79.4)

*LNR* lymph node ratio

## Clinico-pathological characteristics and LNR associations in metastatic colon and patients rectal cancer (Tables 3, 4 and 5)

**Table 3:** Comparison of clinicopathological factors and LNR (stage IV)

	Lymph node ratio			
	Colon (*n* = 339)		Rectum (*n* = 125)	
	β (95% CI)	*P*-value	β (95% CI)	*P*-value
Gender				
Male	Reference	–	Reference	–
Female	0.004(−0.05 to 0.06)	0.89	–0.004(−0.10 to 0.09)	0.93
Age (mean [SD]), years	−0.001(−0.003 to 0.001)	0.39	0.0001(−0.004 to 0.003)	0.87
BMI (mean [SD]), kg/m^2^	0.0001(−0.01 to 0.01)	0.88	0.004(−0.01 to 0.02)	0.61
ASA grade	−0.03(-0.07 to 0.01)	0.15	−0.02(−0.09 to 0.05)	0.61
I				
II				
III/IV				
Year of surgery	*−0.02(−0.04 to −0.003)*	*0.02* ^*^	−0.02(–0.05 to 0.01)	0.19
1995–1999 2000–2004 2005 to 2009 2010–2014 2015–2019 2020 onwards				
Emergency operation				
No	Reference	–	Reference	–
Yes	−0.03(−0.10 to 0.03)	0.32	0.07(−0.25 to 0.38)	0.68
Tumour perforation				
No	Reference	–	Reference	–
Yes	−0.05(−0.14 to 0.05)	0.30	0.11(−0.02 to 0.24)	0.09
Obstructive presentation				
No	Reference	–	Reference	–
Yes	−0.04(−0.11 to 0.04)	0.35	0.07(−0.25 to 0.38)	0.68
Operation modality		0.27		
Open	Reference		Reference	–
Laparoscopy	−0.03(−0.09 to 0.03)		−0.09(−0.19 to 0.02)	
Operation type		0.05	†	0.12
Segmental	−0.09(−0.18 to 0.002)			
Standard				
Extended				
Colon cancer location				
Right	*Reference*	*–*		
Left	*−0.07(−0.12 to −0.01)*	*0.02* ^*^		
Rectal cancer location			−0.04(−0.10 to 0.02)	0.17
Upper				
Mid				
Lower				
Neoadjuvant RT (rectal)				
No			Reference	–
Yes			−0.06(−0.19 to 0.08)	0.44
Distant metastatic sites				
Liver or other	*Reference*	*–*	*Reference*	*–*
Liver and other(s)	*0.33(0.24 to 0.42)*	< 0.001^*^	*0.41(0.26 to 0.57)*	< 0.001^*^
Distant mets resected				
No	*Reference*	*–*	*Reference*	*–*
Yes	*−0.09(−0.16 to −0.02)*	*0.02* ^*^	*0.13(−0.25 to −0.02)*	*0.03* ^*^
Tumour size (median [IQR]), cm	0.003(–0.01 to 0.02)	0.67	0.01(–0.02 to 0.04)	0.40
T stage	*0.16(0.07 to 0.17)*	< 0.001^*^	0.09(−0.01 to 0.18)	0.07
T1 and T2				
T3				
T4				
Histological type				
Mucinous/signet ring	Reference	–	*Reference*	–
Non-mucinous/signet ring	−0.05(–0.14 to 0.03)	0.22	*−0.26(–0.45 to –0.07)*	*0.008* ^*^

*BMI* body mass index, *ASA* American Society of Anesthesiology, *RT* radiotherapy* T stage* tumour stage (part of AJCC TNM system); † All stage IV rectal cancers were managed with a standard resection; * *P* < 0.05

**Table 4:** Comparison of clinicopathological factors between patients with LNR of 0 and LNR > 0 in stage IV colon cancer (*n* = 339)

Variables	*N* (%) or mean (SD) or median (range/ IQR)	LNR of 0 *n* (%) (Total of 66)	LNR > 0 *n* (%) (Total of 273)	*P* value	Odds ratio (95% CI)
Gender					
Male	191(56.3)	41(62.1)	150(54.9)	–	Reference
Female	148(43.7)	25(37.9)	123(45.1)	0.29	1.35(0.78–2.33)
Age (mean [SD]), years	69.1(13.6)	74.0(11.1)	67.9(13.9)	*0.001* ^*^	*0.96(0.94–0.99)*
BMI (mean [SD]), kg/m^2^	27.1(5.7)	26.5(4.9)	27.3(5.8)	0.50	1.03(0.95–1.11)
ASA grade				0.12	0.72(0.47–1.09)
I	40(11.8)	7(10.6)	33(12.1)		
II	164(48.4)	26(39.4)	138(50.5)		
III/IV	135(39.8)	33(50.0)	102(37.4)		
Year of surgery				0.47	1.07(0.89–1.28)
1995–1999	61(18.0)	14(21.2)	47(17.2)		
2000–2004	59(17.4)	9(13.6)	50(`8.3)		
2005–2009	68(20.1)	19(28.8)	49(17.9)		
2010–2014	71(20.9)	10(15.2)	61(22.3)		
2015–2019	64(18.9)	10(15.2)	54(19.8)		
2020 onwards	16(4.7)	4(6.1)	12(4.4)		
Emergency operation			219(80.2)	–	Reference
No	265(78.2)	46(69.7)	54(19.8)	0.07	0.57(0.31
Yes	74(21.8)	20(30.3)			–1.04)
Tumour perforation					
No	307(90.6)	58(87.9)	249(91.2)	–	Reference
Yes	31(9.1)	8(12.1)	23(8.8)	0.41	0.70(0.30–1.63)
Obstructive presentation					
No	280(82.6)	52(78.8)	228(83.5)	–	Reference
Yes	59(17.4)	14(21.2)	45(16.5)	0.37	0.73(0.38–1.43)
Operation modality					
Open	229(67.6)	47(71.2)	182(66.7)		Reference
Laparoscopy	110(32.4)	19(28.8)	91(33.3)	0.48	1.24(0.69–2.23)
Operation type	3(0.9)	1(1.5)	2(0.7)	0.31	1.68(0.62–4.55)
Segmental	306(90.	61(92.4)	245(89.7)		
Standard	3)	4(6.1)	26(9.5)		
Extended	30(8.8)				
Colon cancer location					
Right	145(42.8)	22(33.3)	123(45.1)	–	Reference
Left	194(57.2)	44(66.7)	150(54.9)	0.09	0.61(0.35–1.07)
Distant metastatic sites					
Liver or other	279(82.3)	59(89.4)	220(80.5)	*–*	*Reference*
Liver and other(s)	54(15.9)	5(7.6)	49(17.9)	*0.049* ^*^	*2.63(1.002–6.89)*
Distant mets resected					
No	275(81.1)	57(86.4)	218(79.9)		Reference
Yes	58(17.1)	7(10.6)	51(18.7)	0.13	1.91(0.82–4.42)
Tumour size (median [IQR]), cm	5.0(4.0–6.0)	5.5(4.1–7.3)	5.0(4.0–6.0)	*0.02* ^*^	*0.87(0.78–0.98)*
T stage				*0.02* ^*^	*2.24(1.33–3.77)*
T1 and T2	6(1.8)	1(1.5)	5(1.8)		
T3	173(51.0)	46(69.7)	127(46.5)		
T4	160(47.2)	19(28.8)	141(51.6)		
Histological type				0.86	1.08(0.47–2.47)
Mucinous/sig net ring	39(11.5)	8(12.1)	31(11.4)		
Non-mucinous/signet ring	300(88.5)	58(87.9)	242(88.6)		

*BMI* body mass index, *ASA* American Society of Anesthesiology, *T stage* tumour stage (part of AJCC TNM system); * P < *0.05*

**Table 5:** Comparison of clinicopathological factors between patients with LNR = 0 and LNR > 0 in stage IV rectal cancer (*n* = 125)

Variables	*N* (%) or mean (SD) or median (range/ IQR)	LNR = 0 *n* (%) (Total of 18)	LNR > 0 *n* (%) (Total of 107)	*P* value	Odds ratio (95% CI)
Gender					
Male	78(62.4)	9(50.0)	69(64.5)	–	Reference
Female	47(37.6)	9(50.0)	38(35.5)	0.89	0.92(0.30–2.82)
Age (mean [SD]), years	65.4(12.6)	66.8(12.8)	65.1(12.6)	0.61	0.99(0.95–1.03)
BMI (mean [SD]), kg/m^2^	27.0(5.0)	28.0(6.8)	26.9(4.7)	0.61	0.96(0.81–1.14)
ASA grade				0.49	0.77(0.36–1.62)
I	28(22.4)	2(11.1)	26(24.3)		
II	68(54.4)	12(66.7)	56(52.3)		
III/IV	29(23.2)	4(22.2)	25(23.4)		
Year of surgery				0.50	1.13(0.79–1.62)
1995–1999	37(29.6)	8(44.4)	26(24.3)		
2000–2004	36(28.8)	4(22.2)	32(29.9)		
2005–2009	26(20.8)	1(5.6)	25(23.4)		
2010–2014	11(8.8)	2(11.1)	9(8.4)		
2015–2019	10(8.0)	2(11.1)	8(7.5)		
2020 onwards	8(6.4)	1(5.6)	7(6.5)		
Emergency operation					
No	122(97.6)	18(100.0)	104(97.2)		
Yes	3(2.4)	–	3(2.8)		
Tumour perforation					
No	106(84.8)	17(94.4)	89(83.N2)	–	Reference
Yes	19(5.2)	1(5.6)	18(16.8)	0.24	3.44(0.43–27.51)
Obstructive presentation					
No	122(97.6)	18(100.0)	104(97.2)	–	Reference
Yes	3(2.4)	–	3(2.8)	1.00	NA
Operation modality					
Open	92(73.6)	13(72.2)	79(73.8)	–	Reference
Laparoscopy	33(26.4)	5(27.8)	28(26.2)	0.89	0.92(0.30–2.82)
Operation type		–	–		
Segmental	–	–	–		
Standard	125(100.0)	18(100.0)	107(100.0)		
Extended	–	–	–	–	
Rectal cancer location				*0.02* ^*^	*0.45(0.23–0.87)*
Upper	47(37.6)	3(16.7)	44(41.1)		
Mid	43(34.4)	6(33.3)	37(34.6)		
Lower	35(28.0)	9(50.0)	26(24.3)		
Neoadjuvant RT (rectal)					
No	108(86.4)	12(66.7)	96(89.7)	*–*	*Reference*
Yes	17(3.6)	6(33.3)	11(10.3)	*0.01* ^*^	*0.23(0.07–0.73)*
Distant metastatic sites					
Liver or other	103(82.4)	18(100.0)	85(79.4)		Reference
Liver and other(s)	21(16.8)	–	21(19.6)	NA	NA
Distant mets resected					
No	99(79.2)	12(66.7)	87(81.3)	–	Reference
Yes	25(20.0)	6(33.3)	19(17.8)	0.14	0.44(0.15–1.31)
Tumour size (median [IQR]), cm	5.0(4.0–6.0)	5.3(4.5–7.1)	5.0(4.0–6.0)	0.35	0.88(0.66–1.16)
T stage				*0.03* ^*^	*6.55(1.8*
T1 and T2	7(5.6)	3(16.7)	4(3.7)		*6–23.09)*
T3	89(71.2)	15(83.3)	74(69.2)		
T4	29(23.2)	–	29(27.1)		
Histological type					
Mucinous/signet ring	8(6.4)	–	8(7.5)	–	Reference
Non-mucinous/signet ring	117(93.6)	18(100.0)	99(92.5)	NA	NA

*BMI* body mass index, *ASA* American Society of Anesthesiology, *RT* radiotherapy, *T stage* tumour stage (part of AJCC TNM system); ^*^ *P* < 0.05

The associations between standard clinico-pathological factors and LNR are summarised in Table 3 (LNR as a continuous variable), Table 4 and 5 (LNR as a dichotomous variable).

### Colon cancer

- (1) LNR (continuous): On linear regression, LNR was significantly associated with the year of resection (β −0.02 [95% CI −0.04 to −0.003]; *P*=0.02), left-sided colon cancers (β −0.07 [95% CI −0.12 to −0.01]; *P*=0.02), number of metastatic sites (β 0.33[95% CI 0.24–0.42]; *P*<0.001), distant metastasectomy (β −0.09 [95% CI −0.16 to −0.02]; *P*=0.02) and depth of tumour invasion (β 0.16 [95% CI 0.07–0.17]; *P*<0.001)
- (2) LNR (dichotomised): On logistic regression, LNR>0 was associated with age (OR 0.96 [95% CI 0.94, 0.99]; *P*=0.001), number of metastatic sites (OR 2.63 [95% CI 1.002, 6.89]; *P*=0.049), tumour size (OR 0.87 [95% CI 0.78, 0.98]; *P*=0.02) and depth of tumour invasion (OR 2.24 [95% CI 1.33, 3.77];*P*=0.002).

### Rectal cancer

- (1) LNR (continuous): On linear regression, LNR was significantly associated with number of metastatic sites (β 0.41[95% CI 0.26, 0.57]; *P*<0.001), distant metastasectomy (β −0.13[95% CI −0.25, −0.02]; *P*=0.03) and histology type (β −0.26[95% CI -0.45, −0.07]; *P*=0.008).
- (2) LNR (dichotomised): On logistic regression, LNR>0 was associated with decreasing rectal tumour height (OR 0.45 [95% CI 0.23, 0.87]; *P*=0.02), neo-adjuvant (chemo)radiotherapy (OR 0.23 [95% CI 0.07, 0.73]; *P*=0.01), and depth of tumour invasion (OR 6.55 [95% CI 1.86, 23.09]; *P*=0.003).

### Comparison of survival outcomes between clinico-pathological characteristics including LNR in patients with metastatic colon and rectal cancer (Table 6 and Table 7)

**Table 6:** Clinicopathological Features and Oncological Associations stage IV (*n* = 464) – LNR CONTINUOUS

	Overall survival								
	Colon					Rectum			
	HR (95% CI)		*P* value	aHR (95% CI)	*P* value	HR (95% CI)	*P* value	aHR (95% CI)	*P* value
Year of surgery 1995–1999 2000–2004 2005–2009 2010–2014 2015–2019 2020 onwards	*0.84(0.77–0.91)*		< 0.001^*^	*0.84(0.77–0.92)*	< 0.001^*^				
Colon cancer location									
Right	*Reference*			*Reference*	*–*				
Left	*0.68(0.54–0.86)*		*−0.001* ^*^	*0.68(0.54–0.87)*	*0.002* ^*^				
Distant metastatic sites									
Liver or other	*Reference*		^*-*^	Reference	–	Reference	–	Reference	–
Liver and other(s)	*1.52(1.12–2.06)*		*0.007*	1.24(0.90–1.71)	0.18	1.34(0.81–2.22)	0.26	0.82(0.48–1.40)	0.46
Distant mets resected									
No	*Referencere*		*–*	*Reference*	*–*	*Reference*	*–*	*Reference*	*–*
Yes	*0.36(0.25–0.50)*		< 0.001^*^	*0.43(0.30–0.62)*	< 0.001^*^	*0.36(0.22–0.60)*	< 0.001^*^	*0.39(0.23–0.65)*	< 0.001^*^
T Stage T1 and T2 T3 T4	*1.42(1.14–1.76)*		*0.02* ^*^	*1.41(1.11–2.68)*	*0.006* ^*^				
Histological type									
Mucinous/signet ring									
						*Reference*	–	*Reference*	–
Non-mucinous/signet ring						0.80(0.39–1.64)	0.54	0.95(0.46–1.98)	0.90
LNR (continuous)	*2.72(1.78–4.16)*	< 0.001^*^		*1.72(1.11–2.68)*	*0.02* ^*^	*3.83(1.98–7.40)*	< 0.001^*^	*3.48(1.76–6.88)*	< 0.001^*^

*HR* hazard ratio, *aHR* adjusted hazard ratio, *RT* radiotherapy, *LNR* lymph node ratio; * *P* < 0.05

**Table 7:** Clinicopathological Features and Oncological Associations stage IV (*n* = 464) – LNR CATEGORISED

	Overall survival							
	Colon				Rectum			
	HR (95% CI)	*P* value	aHR (95% CI)	*P* value	HR (95% CI)	*P* value	aHR (95% CI)	*P* value
Age, years	*1.02(1.01–1.03)*	< 0.001^*^	*1.02(1.02–1.03)*	< 0.001^*^				
Rectal cancer location					1.00(0.79–1.26)	0.99	1.09(0.84–1.43)	0.52
Upper								
Mid								
Lower								
Neoadjuvant RT (rectal)								
No					*Reference*	–	*Reference*	–
Yes					0.64(0.34–1.20)	−0.17	0.77(0.40–1.49)	−0.43
Distant metastatic sites								
Liver or other	*Reference*	*–*	*Reference*	*–*				
Liver and other(s)	*1.52(1.12–2.06)*	*−0.0*	*1.42(1.04–1.94)*	*−0.03*				
Tumour size, cm	1.01(0.96–1.07)	*07* ^*^	1.00(0.95–1.06)	0.91				
T stage T1 and T2 T3 T4	*1.42(1.14–1.76)*	*0.002* ^*^	*1.41(1.12–1.77)*	*0.003* ^*^	1.08(0.73–1.58)	0.71	0.94(0.60–1.48)	0.80
LNR (Categorised)								
LNR > 0	*Reference*	*–*	*Reference*	*–*	*Reference*	*–*	*Reference*	
LNR of 0	*1.44(1.06–1.96)*	*0.02* ^*^	*1.50(1.08–2.07)*	*0.02* ^*^	*2.21(1.21–4.06)*	*0.01* ^*^	*2.21(1.16–4.24)*	*0.02* ^*^

*HR* hazard ratio, *aHR* adjusted hazard ratio, *RT* radiotherapy, *LNR* lymph node ratio; * *P* < 0.05

**Fig. 2:**
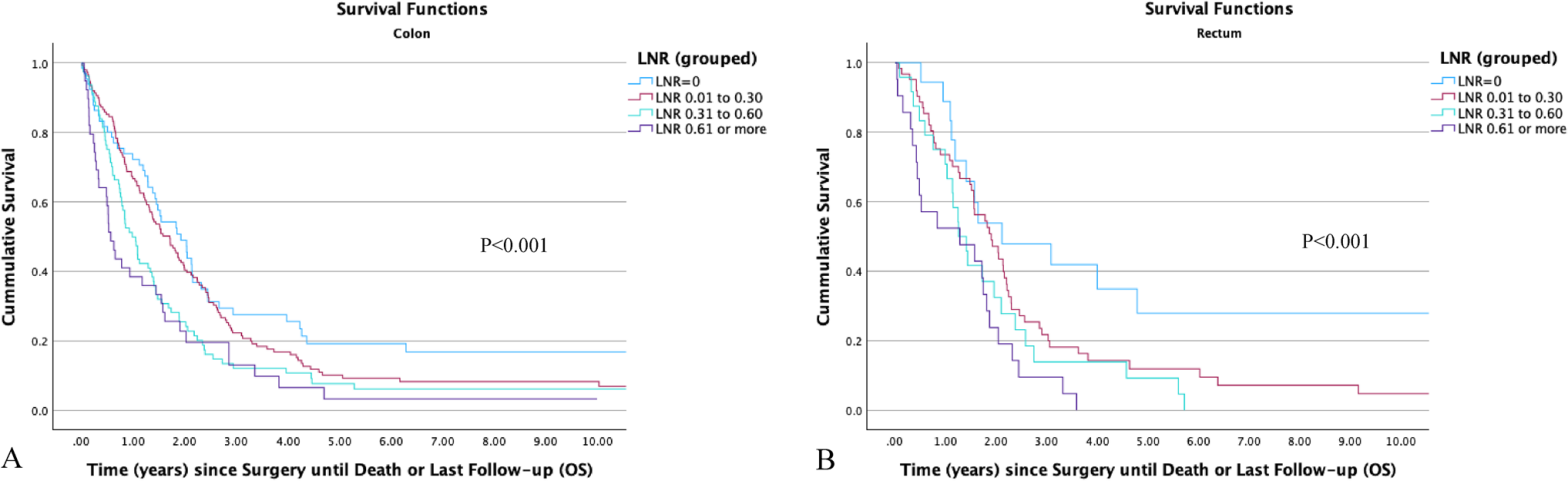
Kaplan–Meier plots of OS of patients with stage IV colon (**A**) and rectal (**B**) cancer stratified by LNRs

**Fig. 3:**
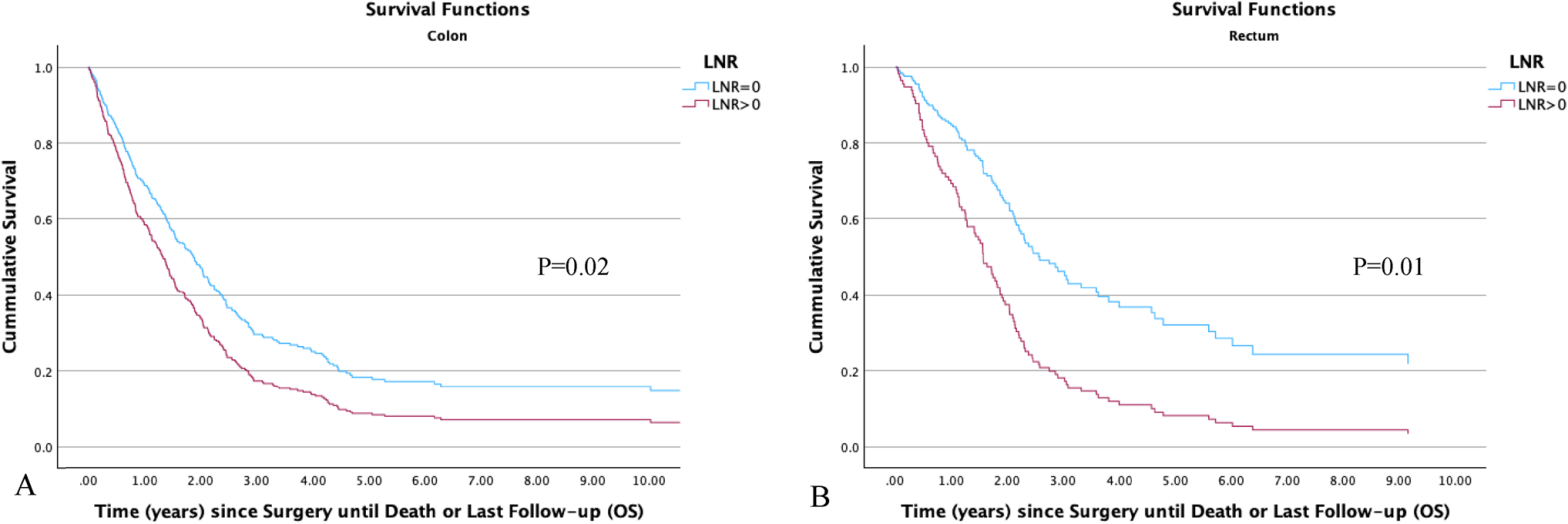
Kaplan–Meier plots of OS of patients with stage IV colon (**A**) and rectal (**B**) cancer stratified by LNR of 0 and LNR > 0

The associations between LNR and OS for both colon and rectal cancer patients, are presented in Table 6 and  7. Additionally, Fig. 2 and Fig. 3 present the Kaplan–Meier plots illustrating the OS for these patients.

### Colon cancer

Death occurred in 289 colon cancer patients (85.3%), primarily due to CRC, with only 34 patients (11.8%) succumbing to non-cancer causes. The 5 year OS was 10.5% (95% CI 8.7–12.3).

- (1) LNR (continuous): on multivariate analysis with LNR as a continuous variable, increasing LNR (*P*=0.02) and T stage (*P* = 0.006) correlated with poorer survival. Conversely, improved OS was associated with later years of the study (*P*<0.001), left-sided tumours (*P* = 0.002) and distant metastasectomy (*P*<0.001).
- (2) LNR (dichotomised): when analysed as a categorical variable in a multivariate model, LNR>0 was associated with poorer survival (*P* = 0.02), as was increasing age (*P*<0.001), number of metastatic sites (*P* = 0.03) and increasing depth of tumour invasion (*P*=0.003).

### Rectal cancer

In patients with rectal cancer, 109 individuals (87.2%) died, with 103 (94.5%) deaths attributed to rectal cancer and six to other causes. The 5 year OS rate was 11.5% (95% CI 8.4–14.6).

- (1) LNR (continuous): In a multivariate model, poorer survival was noted with increasing LNR (*P* < 0.001), while improved survival was observed in those undergoing a distant metastasectomy (*P* < 0.001).
- (2) LNR (dichotomised): On multivariate analysis, only LNR > 0 was associated with poorer survival (*P*=0.02).

## Discussion

This extensive 27 year cohort study aimed to investigate the prognostic significance of LNR in patients with stage IV CRC who underwent upfront resection of their primary tumour. The relationship between LNR and OS was tested (adjusting for confounders of LNR), treating LNR as both a continuous and dichotomous variable. In both colon and rectal cancer patients, an increasing LNR was found to worsen OS. Moreover, patients with mCRC who exclusively had non-lymphatic routes of dissemination (i.e. LNR of 0 or N0 status) displayed a more favourable prognosis than those with lymphatic spread (i.e. LNR > 0).

The significance of LNR as a prognostic factor in mCRC has been previously investigated but with varying outcome measures and study populations [1, 2, 4, 24–30]. Most studies, similar to ours, have confirmed an association between increasing LNR and poorer prognosis [1, 2, 4, 24–32]. Some, however, included patients with stage I/II CRC in their study cohorts, wherein defining an LNR would seem inappropriate (given these patients all have an LNR of 0, by definition). This approach may skew the nodal metastasis and survival outcome data [28, 30]. Additionally, a few studies investigated only colon cancer patients [2, 27], focussed solely on either palliative resection [1] or curative resection [4, 29] and violated principles of collinearity [24, 32]. Notwithstanding this, our study adds to a growing body of literature that promotes the prognostic importance of LNR and its valuable contribution to patient survival following resection of the primary tumour, even in patients with stage IV CRC.

This study uniquely dichotomised patients into those with an LNR of 0, and those with LNR > 0. To date, limited studies have explored survival differences based on this LNR dichotomization [3, 33]. In the study by Zhang et al. [3], LNR was stratified into five groups, including LNR of 0, to validate the discriminatory performance of previously published LNR cut-offs by Rosenberg et al., [33]. However, in both studies, the large number of LNR of 0 patients were confounded by the inclusion of patients with early-stage CRC. Dichotomisation of patients into those with an LNR of 0 and LNR > 0 is more meaningful, as it groups tumours based on their observed routes of spread. Specifically, patients with an LNR of 0 represent those with tumours that have spread exclusively via haematogenous routes and/or transcoelomic spread but not by lymphatic spread. By contrast, tumours with an LNR > 0 are those demonstrating potentially all three routes of dissemination. Intuitively, the absence of LN spread in the presence of haematogenous or transcoelomic spread, as observed in LNR of 0 patients, may identify patients whose tumours have potentially favourable biological behaviour and decreased tumour burden. Indeed, in our study, LNR of 0 served as an independent predictor of improved survival in patients with mCRC.

Notably, the majority of patients in our cohort had at least twelve LNs harvested during surgery. This probably reflects a standardised surgical approach to performing a CRC resection in our unit, irrespective of the presence of distant metastases. This approach is not universally embraced, though, as some surgeons argue that the lymphadenectomy holds little prognostic value for a mCRC resection as the presence of distant metastases overrides prognostication in these circumstances [5, 34]. Nevertheless, a separate study by Jiao et al. demonstrated improved survival in patients with mCRC with at least twelve LNs harvested, highlighting the role of an oncologically adequate lymphadenectomy as an element of good quality mCRC surgery [11]. Building on this, the demonstrable prognostic importance of LNR (specifically LNR of 0 or N0 status) as demonstrated in our study can only be reliably ascertained in the presence of an adequate lymphadenectomy (i.e., LNY of at least twelve nodes).

The finding of a relatively small proportion of patients (16.6%) in our series who underwent an urgent operation warrants discussion. Published literature suggests that patients requiring urgent surgery represent a population with poorer outcomes compared with those with non-urgently resected CRCs [16]. This disparity in outcomes might stem from complex clinical presentations, such as perforation, obstruction or bleeding, and potentially insufficient LNY, which could lead to imprecise staging [16]. However, contrary to the perception that emergency surgery may be responsible for a substandard oncological resection, this study revealed no significant difference between urgent surgery and LNR, irrespective of its assessment as a continuous or dichotomous variable. In our study, the majority of operations were elective. This approach of elective upfront primary tumour surgery for mCRC remains widely recognised and practised today, underlining the generalisability of our findings [2–4, 11, 13, 14, 25].

The optimal extent of resection and lymphadenectomy for patients with mCRC remains controversial. While segmental resection with limited lymphadenectomy may be considered in some cases, our study does not support its routine adoption. As already mentioned, the significant difference in survival observed between those with exclusively haematogenous or transcoelemic spread (i.e. LNR of 0 or N0 status) and LNR > 0 (where lymphatic routes are also implicated), emphasizes the importance of conducting an adequate lymphadenectomy to accurately ascertain the nodal status of mCRCs. Achieving this may be more feasible with a standard or extended resection compared with segmental resections. While our study was not primarily focused on evaluating the extent of resection in patients with mCRC, it highlights the benefits of performing a standardised approach to CRC resection whenever possible in all patients, regardless of whether the operation is executed laparoscopically or by an open approach. Furthermore, one could argue that standardising surgical techniques across all stages of CRC, regardless of metastatic status, contributes to a safer surgical approach by eliminating the risk of taking ‘shortcuts’.

This study has limitations. The retrospective design introduces inherent biases. The limited rate of distant metastasectomy and the omission of patients who underwent nCT reduces our study population and narrows the generalizability of our findings. Likewise, the use of neo-adjuvant (chemo) radiotherapy in patients with rectal cancer may have influenced LNY and nodal positivity rates. Whilst only 13% of patients received neo-adjuvant (chemo) radiotherapy, selection was primarily based on a patient’s individual needs in accordance with contemporary practice guidelines at the time. Moreover, while our study primarily focused on patients with mCRC who received upfront primary tumour resection – a recognized treatment strategy [13, 14] – the uptake of nCT and liver-first surgery in managing mCRC may limit this study’s generalizability to other treatment paradigms [35]. Finally, the limited availability of tumour biomarkers (e.g. BRAF, NRAS) restricts our analysis and comprehensive understanding of the biological behaviour of the tumour in patients with mCRC.

This study enhances our understanding of mCRC management by investigating the prognostic value of the LNR in patients who underwent upfront surgery for the primary tumour. Notably, this study stands out as the only cohort study to date that has specifically examined the survival outcomes using dichotomization of LNR into LNR of 0 and LNR > 0. Our results highlight that this dichotomization holds useful prognostic information, with LNR of 0 (i.e. N0 status) patients identified as a unique subgroup with a better prognosis. Thus, rather than dismissing it as futile, we emphasize the importance of conducting an adequate lymphadenectomy (i.e. harvesting at least 12 LNs) to accurately stage patients with mCRC. Furthermore, identifying LNR of 0 patients as a distinct subgroup calls for further research into their molecular and genetic characteristics, paving the way for refined, individualized treatment approaches and targeted therapies in the management of mCRC.

## Data Availability

The data that support the findings of this study are available from the corresponding author, Dr Kheng-Seong Ng, upon reasonable request.
